# Lipid–Polymer Hybrid Nanosystems: A Rational Fusion for Advanced Therapeutic Delivery

**DOI:** 10.3390/jfb14090437

**Published:** 2023-08-23

**Authors:** Shweta Jain, Mudit Kumar, Pushpendra Kumar, Jyoti Verma, Jessica M. Rosenholm, Kuldeep K. Bansal, Ankur Vaidya

**Affiliations:** 1 Sir Madan Lal Institute of Pharmacy, Etawah 206310, India; shwetavaidya2000@gmail.com; 2 Faculty of Pharmacy, Uttar Pradesh University of Medical Sciences, Saifai, Etawah 206130, India; muditcology@gmail.com (M.K.); pushydv15@gmail.com (P.K.); 3 Pharmaceutical Sciences Laboratory, Faculty of Science and Engineering, Åbo Akademi University, 20520 Turku, Finland; jyoti.verma@abo.fi (J.V.); jessica.rosenholm@abo.fi (J.M.R.)

**Keywords:** lipid–polymer hybrid nanoparticles, cancer, drug delivery, synthesis, release mechanism

## Abstract

Lipid nanoparticles (LNPs) are spherical vesicles composed of ionizable lipids that are neutral at physiological pH. Despite their benefits, unmodified LNP drug delivery systems have substantial drawbacks, including a lack of targeted selectivity, a short blood circulation period, and in vivo instability. lipid–polymer hybrid nanoparticles (LPHNPs) are the next generation of nanoparticles, having the combined benefits of polymeric nanoparticles and liposomes. LPHNPs are being prepared from both natural and synthetic polymers with various techniques, including one- or two-step methods, emulsification solvent evaporation (ESE) method, and the nanoprecipitation method. Varieties of LPHNPs, including monolithic hybrid nanoparticles, core–shell nanoparticles, hollow core–shell nanoparticles, biomimetic lipid–polymer hybrid nanoparticles, and polymer-caged liposomes, have been investigated for various drug delivery applications. However, core–shell nanoparticles having a polymeric core surrounded by a highly biocompatible lipid shell are the most commonly explored LPHNPs for the treatment of various diseases. In this review, we will shed light on the composition, methods of preparation, classification, surface functionalization, release mechanism, advantages and disadvantages, patents, and clinical trials of LPHNPs, with an emphasis on core–shell-structured LPHNPs.

## 1. Introduction

Nanoparticles (NPs) made of either polymers or lipids are widely used for the delivery of therapeutic agents. Polymeric nanocarriers are chiefly categorized as polymeric micelles, polymer–drug conjugates, and polymeric NPs, while lipid-based nanocarriers are distinguished as liposomes, solid lipid nanoparticles (SLNs), and nanostructured lipid vectors [1,2]. Polymeric nanoparticles (PNPs) are currently gaining popularity in biomedical research, possibly due to their numerous attractive properties, including biocompatibility, biodegradability, the ease of processing, and sustained release patterns of incorporated drugs [3]. PNPs are easy to prepare with simple and reproducible synthesis processes, with ample flexibility for chemical modifications [4]. Polymeric NPs usually have low polydispersity and high stability. However, there are certain limitations associated with using polymers as nanocarriers for drug delivery, for instance, the use of harmful organic solvents in the preparation, the expensive synthesis, the scale-up process, the toxicity of degradation products, and the usually low drug loading capacity [5,6]. Moreover, the hydrophobic surface of PNPs is recognized as a foreign material that provokes an immune response and prompts rapid elimination from the body, which limits their use. Furthermore, the limited cellular membrane permeability of PNPs leads to poor transfection efficiency [7,8]. 

Lipid nanoparticles, similar to PNPs, have piqued the interest of researchers in recent decades and have had remarkable clinical success in delivering drugs, nucleic acids, and vaccines. Lipid nanoparticles offer numerous attractive benefits, including low production costs with an ease of preparation, an improved drug-entrapping efficiency, great biocompatibility, feasibility of scale-up, and targeted delivery. However, they have abridged stability, fast drug release, and excessive polydispersity, which are the major limitations for their widespread use [9,10,11]. 

**Figure 1:**
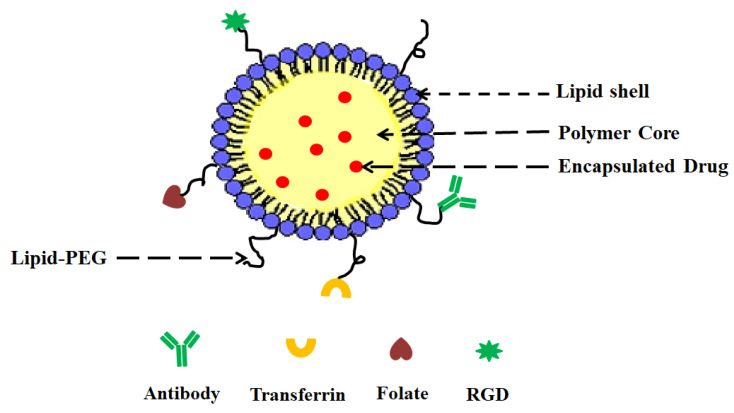
General structure of LPHNP composed of a drug-encapsulating polymer core with an outer lipid shell and an outer lipid–PEG layer.

To overcome these limitations of PNPs and lipid nanoparticles, a new system called lipid–polymer hybrid nanoparticles (LPHNPs) has been developed owing to the biomimetic and biocompatible advantages of this strategy. LPHNPs take advantage of the benefits of both polymeric and lipid-based systems [12]. LPHNPs are next-generation core–shell nanosystems conceptually formed from a polymer core wrapped by a lipid layer. Despite their widespread attention, they are not yet widely used or pervasive [13]. LPHNPs have three structural features, including (Figure 1):

(i) A drug encapsulating polymer core;
(ii) A lipid layer surrounding the polymer core;
(iii) An outer lipid–PEG layer.

The lipid layer acts as a molecular barrier, reducing drug loss during LPHNP formulation and further protecting the core from degradation by blocking water diffusion into the inner core. The outer lipid–PEG layer prolongs the blood circulation of LPHNPs by suppressing the immune response [14]. 

## 2. Advantages and Limitations of LPHNPs

LPHNPs combine the benefits of polymeric nanocarriers, such as favorable drug release profiles and surface chemical functionalization or modification; with the benefits of lipid-based nanocarriers, such as improved drug loading capacity and biocompatibility [15,16,17]. Recent research trends indicate that LPHNPs will, for instance, be extremely helpful or productive in the treatment of diseases such as glioblastoma [18]. LPHNPs offer the advantage of being able to select the polymer for a controlled drug release using stimuli-responsive polymers. The outer lipid layer prevents the inner polymeric materials from leaking and seizes the water penetration from the outside to the inside. Such a framework ensures that the formulation is continuously resilient for maximal structural integrity. The lipid-layer coating improves the biocompatibility and stability of NPS upon systemic administration. In comparison to the individual polymer and lipid particles, the LPHNPs have been reported to have a better and longer in vivo efficacy [19]. Although LPHNPs have advantages over PNPs and liposomes, significant drawbacks reduce their translational rate from bench to bedside. It is challenging to optimize the lipid/polymer ratio within NPs [20]. The change in lipid/polymer (L/P) composition during prolonged storage leads to drug leakage. Since phospholipids form the micellar phase while polymers form the nanoparticle phase, this may lead to an increase in the polydispersity, which may result in destabilization, agglomeration, and phase separation. Hydrophilic drug loading into the LPHNP matrix is often limited, which can be overlooked by using the double emulsification method to improve the drug loading characteristics, but it is a time- and energy-consuming method [21,22]. To avoid phase separation, the affinity of the phospholipid to create a monolayer over the polymeric nanoparticle surface must be considered during the phospholipid and polymer selection process. The bulk synthesis of LPHNPs is currently difficult to scale up; nevertheless, a few attempts have been reported, employing microfluidics or multi-inlet vortex reactors [23]. The higher cost of LPHNPs might be due to additional fabrication steps, and the use of both lipids and polymers could incur patient unacceptance [24]. Because of the use of solvents and excipients during fabrication, the environmental impact of LPHNPs synthesis must be carefully considered. The interaction of LPHNPs with plasma proteins must be assayed to examine the modifications required for the pharmacokinetic and pharmacodynamic profiles. The major barrier to gain approval for LPHNPs for human use is the lack of regulatory guidelines [25].

## 3. LPHNPs Composition and Its Influence

LPHNPs composed of natural, semi-synthetic, and synthetic polymers (i.e., chitosan, PCL, polycaprolactone; PEG, polyethylene glycol; PLA, polylactic acid; PLGA, polylactic-co-glycolic acid; PbAE, poly(β-amino ester); etc.) serve as solid core NPs coated with lipids (i.e., palmitic, myristic, and stearic acids, etc.). The simplest design of LPHNPs has a drug-entrapped hydrophilic or hydrophobic polymer core surrounded by a lipid layer. Lipid shells can be easily modified to obtain the desired surface properties which promotes, for instance, efficient uptake, controlled drug release, and favorable biodistribution. The lipid composition can be easily modified and exploited to expedite the covalent or noncovalent attachment of drugs, ligands, transferrin, antibodies, folic acid, aptamers, and bioactive molecules, including nucleic acids or proteins. Lipid shells have the additional advantage of charged or zwitterionic lipids, which may promote self-assembly of the lipid layers with opposite charges on the polymeric core through electrostatic interactions. Likewise, the uses of the lipophilic core endorse its interaction with the hydrophobic tail domain of the lipids constituting the lipid layer [26]. Table 1 presents the polymers and lipids used in the preparation of LPHNPs.

Polymer aggregation and intrinsic viscosity have a direct influence on the particle size. Higher polymer concentrations yield larger particles, while polymers with a higher intrinsic viscosity produce smaller particles [38]. The surface zeta potential of LPHNPs can be altered by changing the end functional groups on shells (usually PEG). Because surface charges prevent particles from colliding, higher absolute zeta potential values result in more stable NPs in vitro. Dave et al. [39] reported that on increasing the concentration of soy lecithin from 20 mg to 30 mg, the zeta potential of the prepared LPHNPs also increased from 23.4 ± 1.5 mV to 41.5 ± 3.4 mV. Furthermore, the interaction of charge inducer (i.e., stearyl amine, SA) with the lipid surface also alters the zeta potential of formulations. According to immunocompatibility tests, the complement activation is the lowest in hybrid NPs with methoxyl surface groups and the highest in NPs with amine surface groups [40]. An adequate surface charge must be chosen to match the hybrid NPs in vitro stability and in vivo immunocompatibility. The lipid-to-polymer ratio (L/P) had a significant impact on the colloidal stability of hybrid particles. The lipid layer may operate as an electrostatic stabilizer when the lipid-to-polymer L/P ratio is high and the cationic lipid concentration is high. At low L/P and cationic lipid fractions, the partial lipid coating of the polymer core was inadequate to colloidally stabilize the LPHNPs. For example, when the anionic surface of one hybrid molecule core was exposed to the cationic 1,2-dipalmitoyl-3-trimethylammonium-propane, LPHNPs aggregation occurred. Surprisingly, when 1,2-dipalmitoyl-sn-glycero-3-phosphocholine (DPPC) was employed alone, the hybrid NPs were less prone to agglomeration. This is because the DPPC’s zwitterionic structure lowers the possibility of electrostatic interactions [41].

Poly(lactic acid) (PLA) is a biopolymer that has gained more attention because of its both biodegradable and biocompatible nature. Because of its high safety profile, the US Food and Drug Administration (US FDA) has approved it for a variety of purposes. For almost two decades, LP-anchored PLA NPs have been employed to deliver medicines, peptides, and vaccinations [42]. The greater mechanical stability of LPHNPs during storage and in serum is attributed to the polymeric core, which behaves as cytoskeleton and to a PEG-coated lipid layer, which improves blood circulation time in vivo by suppressing the immune system [43]. However, the low cell interaction leads to a poor drug delivery efficiency, which is the major limitation. The problem can be resolved by coating or attaching a site-specific target ligand with an outer lipid layer of NPs to mimic the binding with cell surfaces and deliver the payload. The biomimetic LPHNPs retain both the physicochemical features of the synthetic vehicles and inherit the intrinsic functionalities of the cell membranes [44].

Lipids are amphiphilic or hydrophobic molecules and are present in numerous compounds, including fatty acids, oils, steroids, and waxes. Glycerophospholipids are the most common type of biological membrane component, consisting of a glycerol molecule, a phosphate group (PO_4_^2−^), and two fatty acids. These phospholipids are broadly used for the surface engineering of PNPs. Phospholipids, including phosphatidylcholine, phosphatidylglycerol, phosphatidylinositol, phosphatidylserine, phosphatidylethanolamine, and phosphatidic acid are less stable in nature. Thus, synthetic lipids have been reported through the modification of the polar and nonpolar regions of the phospholipid molecules. Synthetic phospholipids, including PEGylated phospholipids, 1,2-diacyl-P-O-ethylphosphatidylcholine, etc., are cationic, anionic, neutral, and zwitterionic phospholipids and are often used in biomedical engineering [45]. Both natural and synthetic lipids have been used for the surface anchoring of PNPs. Natural or cell-membrane-derived lipids have the advantages of natural cell surface, natural targeting ability, natural immune-evading property, long circulating half-life, and well-controlled tissue distribution. Synthetic lipids have the advantages of biomimetic surface, controlled drug release, stealth properties, extended circulation, and surface flexibility for targeting functionalization [41].

The lipid-based surface functionalization of PLGA NPs is showing encouraging outcomes in the fabrication of PLGA-based nanomedicines due to their fascinating properties of biocompatibility, biodegradability, ease of treatment, and sustained release. The targeting specificity of PLGA-based nanocarriers can be enhanced by surface engineering with various lipids. This may further improve the physicochemical properties of nanocarriers as well as their NP–cell associations, including cellular membrane permeability, immune responses, and prolonged blood circulation. PLGA-based LPHNPs have been reported to be utilized for drug and gene delivery [46].

## 4. LPHNP Fabrication Methods

**Figure 2:**
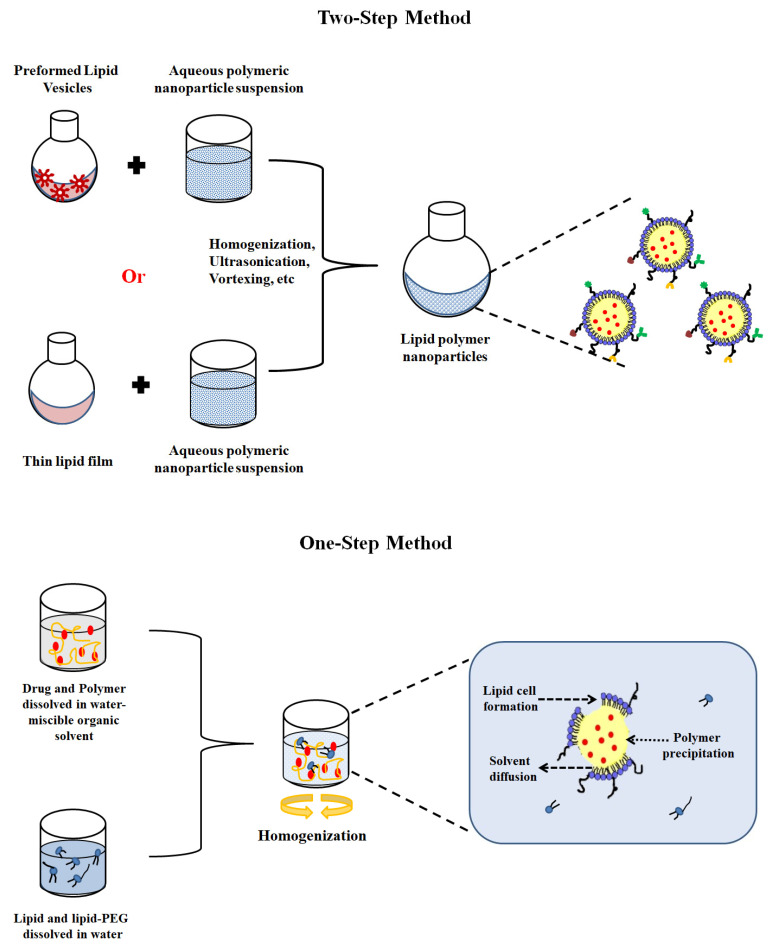
Single- and two-step methods for the preparation of LPHNPs.

LPHNPs have been prepared traditionally by a simple two-step method, but nowadays, a transformation strategy has been introduced that transitions a two-step LPHNPs preparation to a one-step strategy. The latter technique synchronously uses the self-assembly of polymers and lipids. Owing to their two-in-one structure, these LPHNPs are used for combinatorial drug delivery, especially in oncology. The outer lipid–PEG layer can be tailored in such a fashion to achieve the targeting of anticancer therapy, delivery of nucleic acids, and to be used in diagnostic imaging. Although the fusion mechanism of lipid and polymer is still unclear, methods of LPHNP preparation that utilize different mechanisms of formation are presented in Figure 2 [47].

In the two-step method, the LPHNPs are produced by either directly adding the aqueous polymeric nanoparticle suspension to the dried lipid film or first hydrating the thin lipid film with an aqueous solvent to produce lipid vesicles, and then these lipid vesicles are added to an aqueous preformed nanoparticle suspension [48]. In both methods, the hybrids are assembled by the input of external energy provided via vortexing or through the ultrasonication of the suspension and heating at a temperature beyond the phase transition temperature of the lipid constituent. The prepared hybrid structure is thermodynamically stable via electrostatic, hydrophobic, and van der Waals interactions [49].

In the single-step method, the drug and polymer solution in a water-miscible organic solvent is added to an aqueous medium encompassing lipids or lipid–PEG, with spontaneous homogenization, resulting in self-assembly into a monolayer of lipids surrounding the core. During this stage, PEGylated lipids are also self-assembled, with a lipid moiety clinging to the surface of the polymer core and the PEG chain extending externally toward the aqueous environment [50].

The two-step method has the limitation of formulating PNPs and lipid vesicles separately, which requires more energy and is a more time consuming process. The single-step or one-step method is more prevalent and efficient as lipid vesicles and PNPs are not prerequisites in this method. In this method, the spontaneous self-assembled monolayer formation occurs, surrounding the core to form LPHNPs; therefore, it is a rapid and energy-efficient technique [51].

**Figure 3:**
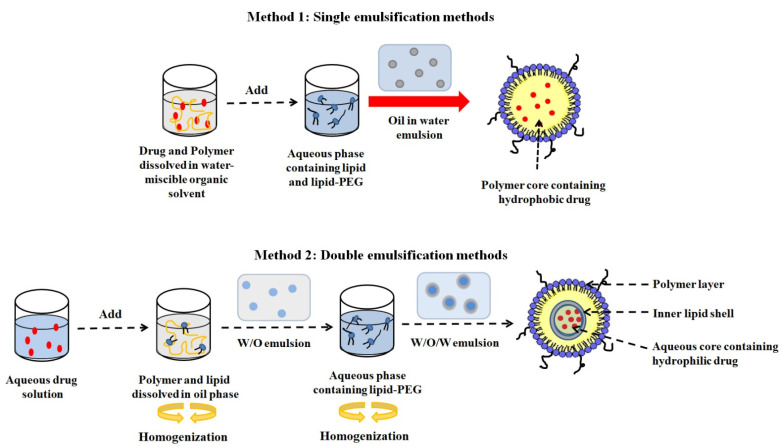
LPHNPs produced by single and double emulsion-solvent evaporation (ESE) methods.

The emulsification solvent evaporation (ESE) method is another technique for the preparation of LPHNPs. This method is further classified into single and double emulsification methods. In the single ESE method, hydrophobic drugs and polymers dissolved in the oil phase are mixed with an aqueous phase containing lipids under constant agitation to form an oil-in-water (o/w) emulsion. The evaporation of the organic medium simultaneously forms a polymer core surrounded by a lipid layer. As an apparent substitute, the lipid can be dissolved in the oil phase alongside the polymer [52]. For water-soluble formulations, the double ESE method is employed to prepare w/o/w emulsion. In this method, the aqueous drug solution is emulsified in an organic solvent that contains polymers and lipids to form a w/o mixture. The prepared w/o mixture was further emulsified in a lipid–PEG-containing aqueous phase to form a w/o/w emulsion, followed by subsequent oil phase evaporation to produce LPHNPs (Figure 3) [53]. The LPHNPs produced by the double ESE method have certain structural anomalies, including: (1) aqueous core surrounded by a lipid layer; (2) a polymer layer in between; and (3) an outer lipid–PEG shell.

On the basis of the desired biomedical applications of LPHNPs, a lipid-based surface engineering method is to be chosen, which further depends on the nature of lipid–polymer surface chemistry. For example, the single-step ESE method is used for gene delivery applications, while the conventional two-step top-down approach is required for nanoghost-based surface engineering [54].

Nanoprecipitation technique is another method for the preparation of LPHNPs. This technique utilizes the dispersion of lipids in the aqueous phase followed by the addition of a drug–polymer solution in a water-miscible organic solvent with sonication, stirring, or homogenization. This causes the polymer to precipitate via solvent diffusion, as well as the lipid to self-assemble onto the polymeric nanoparticle interface [55]. The nanoprecipitation approach was used to prepared triptolide (TL)- and paclitaxel (PTX)-loaded LPHNPs for lung cancer treatment [56]. Prepared LPHNPs were approximately 160 nm in size with a 0.2 polydispersity index. Both drugs showed a greater encapsulating efficiency of about 85%, and the in vivo results revealed around a four-fold reduction in tumor volume as compared with the control group (i.e., PTX and TL). The findings indicate synergy as well as a greater efficiency in treating lung cancer. To achieve a homogenous liquid crystalline phase, the temperature must be kept above the gel-to-sol temperature of lipids during the synthesis. The lipid–aqueous phase acts as an anti-solvent (above their transition temperature) toward a dropwise injection of polymer in organic solvent, resulting in the polymer to aggregate and coil, followed by a self-assembly of lipids surrounding the polymeric core. 

Lipid/polymer ratio plays an imperative role in the synthesis of LPHNPs. As the phospholipid concentration rises over the critical micelle concentration, liposomes form, while decreasing the L/P ratio causes agglomeration due to the anti-solvent impact of the aqueous phase, which reduces lipid content for interfacial stabilization. Furthermore, the use of PEGylated lipids increases the colloidal stability of hybrid NPs. Because of the steric stabilization provided by PEG chains, the PEGylated lipid increases stability without interfering with drug loading and release.

Numerous LPHNP studies are listed in Table 2, describing the preparation methods of LPHNPs, encapsulating materials, targeting ligands, physicochemical properties, and their applications.

**Figure 4:**
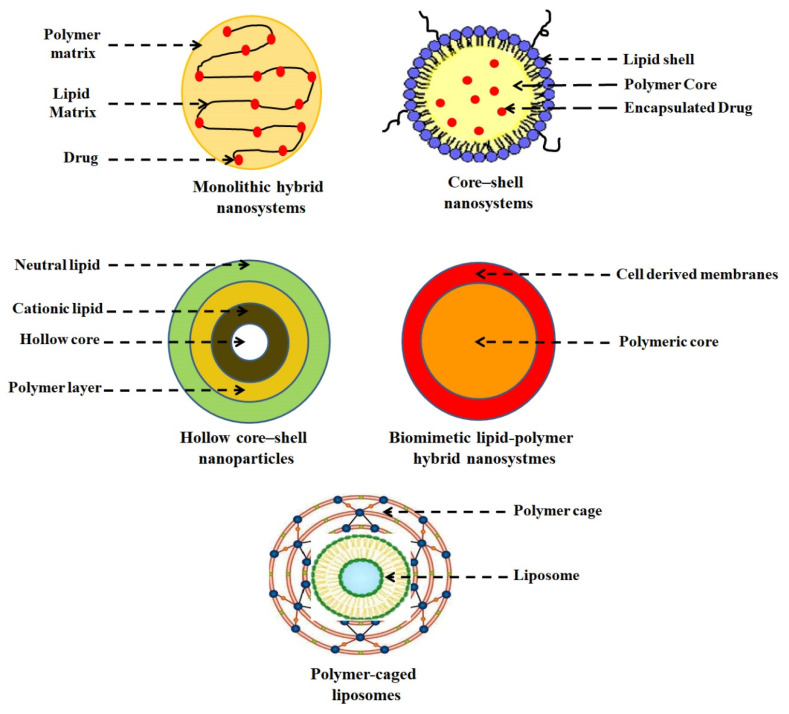
A graphical representation of different types of LPHNPs.

On the basis of structure differences, LPHNPs can be classified as: (i) monolithic hybrid nanosystems, having a polymeric matrix in which lipid molecules are dispersed randomly [44]; (ii) core–shell nanosystems, having a polymeric core surrounded by a highly biocompatible lipid shell [109]; (iii) hollow core–shell NPs, having a hollow inner aqueous core surrounded by an inner polymer layer and cationic and neutral lipid layers [110]; (iv) biomimetic lipid–polymer hybrid nanosystems, having a polymer layer anchored on the surface of liposomes [111]; and (v) polymer-caged liposomes, having a polymer layer surrounded by a biomimetic (Figure 4).

## 5. Surface Functionalization of LPHNPs

**Figure 5:**
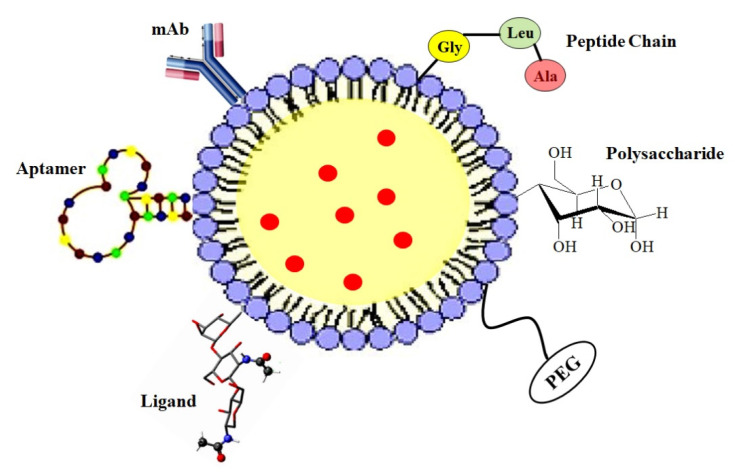
The representation of different surface-anchoring possibilities with LPHNPs.

Surface anchoring is an important and crucial aspect for ameliorating the pharmacokinetics (PKs) and pharmacodynamics (PDs) of LPHNPs. PEGylation of lipids (PEG-lipids) on the outer shell improves blood circulation time but diminishes cell internalization [112,113]. Changing the surface charge, zeta potential, and lipophilicity alters the cell uptake of LPHNPs as well as alters the PK-PD profile of numerous drugs. Surface functionalization imparts target-specific intracellular localization and reduces toxicity. Surface functionalization can be achieved by tethering small molecules, antibodies, cell-penetrating peptides, and aptamers (Figure 5).

Clawson et al. [114] prepared pH-triggered PEG-shedding LPHNPs. To make the LPHNPs pH sensitive, a lipid-(succinate)-methoxy PEG (mPEG) conjugate was prepared that was highly sensitive to acidic hydrolysis and provided a hydrolyzable PEG stealth layer. The lipid-(succinate)-mPEG conjugate hydrolyzes via diester succinate. The pH sensitivity of the NPs can be adjusted by changing the molar concentration of lipid-(succinate)-mPEG in the lipid shell. The more lipid-(succinate)-mPEG integrated into the particle lipid shell, the more stable the particles are at low pH. Thus, by altering the PEG coating, LPHNPs can be tuned over a wide pH range and this concept can be utilized to add potential functionality to the LPHNPs drug delivery toolkit.

Numerous ligands or targeted moieties improve cellular uptake via receptor-mediated endocytosis within target cells. Folic acid (FA) is widely used as a targeting ligand as it evades the attacks of drugs on normal tissues. FA binds firmly to folate receptors (FRs), which are overexpressed in several human tumors. Wu and colleagues [70] improve the efficiency of LPHNPs by introducing FA onto the surface of LPHNPs and forming folate-targeted lipid–polymer hybrid nanoparticles (FLPNPs). The FLPNPs comprise a PLGA core, a lecithin monolayer, a monomethoxy-poly(ethylene glycol)-S-S-hexadecyl (mPEG-S-S-C16) reduction-sensitive shell, and a covalently bound folic acid ligand. The prepared FLPNPs were used for selectively targeted delivery of doxorubicin (DOX) which suppressed human oral cavity squamous cell carcinoma (KB) cell growth and reduced the toxicity of DOX. FLPNPs showed good stability with rapid disassembly in a simulated cancer cell-reductive environment. Furthermore, an increased DOX accumulation in the solid tumor was reported in an animal tumor model by subcutaneously injecting KB cells (1 × 107 per animal) into the flank region of mice. 

In another study, FA-decorated pH-sensitive LPHNPs were utilized for the co-delivery of carboplatin (CBP) and PTX for the treatment of cervical cancer [115]. The formed FA-CBP/PTX-LPNs showed 169.9 ± 5.6 nm particle sizes with a narrow size range of 0.151 ± 0.023. High cellular uptake (66.7 ± 3.1%) with noticeable cell inhibition capacity (23 ± 1.1%) was reported with FA-CBP/PTX-LPNs. Furthermore, FA-CBP/PTX-LPNs revealed pH-sensitive drug release and produced synergistic effects of CBP and PTX on cervical cancer.

Fucose-conjugated nanocarriers open the way for a cure for breast cancer and urge further research [116]. Garg et al. [94] utilized LPHNPs for the co-delivery of methotrexate and aceclofenac, using fucose as a target ligand against MCF-7 and MDA-MB-231 breast cancer cell lines. Prepared fucose-linked LPHNPs have a small particle size (150 nm) with good encapsulation efficiency (85–90%) and drug loading efficiency (10–12%). An improvement in bioavailability (8–10 folds) and the synergism of combinations of drugs led to superior control over tumor growth in the 7,12-dimethylbenz[a] anthracene (DMBA)-induced breast cancer mouse model.

Lipid–polysaccharide surface-modified hybrid NPs were reported by Omar et al. [117] using the nanoprecipitation method for the brain delivery of rivastigmine (Riv). Dextros-cholic acid (DxC) was used as a polysaccharide for the surface modification of lipid–PLGA NPs. Prepared surface-modified LPNs have a size of 111.6 ± 11.4 nm with 92 ± 1.2% an encapsulation efficiency of Riv. The in vivo investigations on albino rats demonstrated that the surface-modified LPNs penetrated the brain more efficiently and quickly than the medication solution. The surface-modified LPNs showed about a five-times higher blood–brain barrier (BBB) penetration than the drug solution. Additionally, the polysaccharide surface modification further improves brain residence time up to 40 h. These results suggested that the lipid–polysaccharide surface-modified hybrid NPs could circumvent the BBB and are predicted to reduce systemic side effects.

Monoclonal antibodies (mAbs) are also used as targeting ligands because they effectively and safely deliver drugs to desired sites; however, a limitation associated with these mAbs is their large hydrodynamic size, which may lead to a significantly increased nonspecific uptake via the reticuloendothelial system (RES) and reduce the NP circulation time. Furthermore, additional limitations of these mAbs include their nonspecific conjugation to NPs (which may result in hindered binding sites of the mAbs) and particle dimerization. The problem can be resolved through the use of small antibody variants, including minibodies, diabodies, and single-chain variable fragments (svFc). Hu et al. [68] synthesized anti-CEA half-antibodies (hAbs) conjugated to LPHNPs via a single-step nanoprecipitation method. The anti-CEA half-antibodies (hAbs) were produced by reducing the disulfide bonds between the heavy chains of the anti-CEA mAbs with a reducing agent. Prepared anti-CEA half-antibody (hAb)-conjugated LPHNPs were extensively characterized, and targeting abilities against pancreatic cancer cells were assayed. The results revealed that the hAb-conjugated lipid–polymer NPs showed an enhanced cancer-killing effect compared to the corresponding plain or unconjugated NPs. 

Similar to mAbs, aptamer-functionalized LPHNPs are also used for the co-delivery of cytotoxic agents for treating cancer. Chen and colleagues [118] designed and synthesized aptamer-functionalized curcumin (CUR) and cabazitaxel (CTX)-loaded LPHNPs (APT-CUR/CTXLPNs) for the treatment of prostate cancer (PC). The A10-3.2 aptamer (5′-GGGAGGACGAUGCGGAUCA GCCAUGUUUACGUCACUCCU-spacer-NH2-3′ with 2′-fluoropyrimidines) was used in this study. The prepared APT-CUR/CTXLPNs had a mean particle size of 121.3 ± 4.2 nm and a positive surface charge of 23.5 ± 2.6 mV. APT-CUR/CTX LPHNPs showed a sustained release of CUR and CTX with an improved tumor inhibition efficiency. At a drug ratio of 2:5 (CUR:CTX), aptamer-functionalized APT-CUR/CTX LPHNPs demonstrated good cell inhibition ability, considerable tumor accumulation, and extraordinary tumor inhibition activity. 

## 6. Stimuli-Responsive Drug Release from LPHNPs

**Figure 6:**
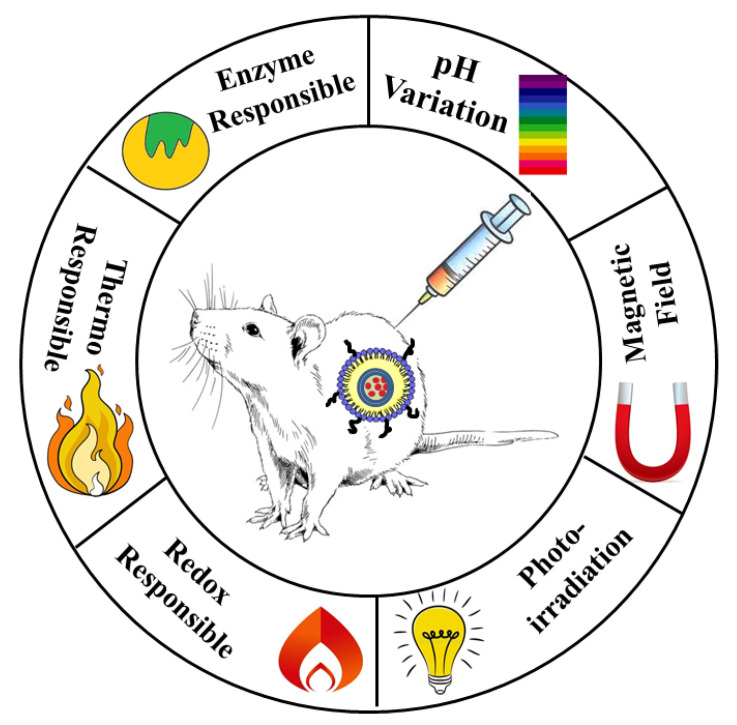
Stimuli-responsive drug release approaches utilized for LPHNPs.

LPHNPs illicit the combined release mechanisms of liposomes and polymeric NPs. Liposomes show drug diffusion and partitioning across the phospholipid bilayer toward the aqueous environment, whereas polymeric NPs exhibit surface and bulk erosion followed by drug diffusion. Further, LPHNPs can be designed for site-specific delivery in response to various stimuli like temperature, pH, redox, and magnetic fields (Figure 6).

For instance, pH-responsive poly(β-amino ester) (PBAE) core–shell NPs coated with lipid polymers (DSPE-PEG2000, FA-DSPE-PEG2000, and lecithin) were prepared (FA/PBAE/DTX-NPs) for the target delivery of docetaxel (DTX) to breast cancer cells, i.e., 4T1 cells. The prepared NPs have uniform particle sizes and excellent physical stability. The in vitro drug release studies showed DTX release on demand at different pHs. The drug release studies at high pH (i.e., pH 7.4 and pH 6.8) showed slower release (60%) as compared to the low pH value (i.e., 90% at pH 5.5). These findings suggested that the acidic environment may stimulate the drug release behavior of FA/PBAE/DTX-NPs within tumor cells. Furthermore, the PBAE tertiary amine group enhanced the endosomal/lysosomal outflow through the proton sponge effect, and ultimately, the rapid release of DTX was observed [119].

Two-component reduction-sensitive LPHNPs (SLPNPs) were prepared from PCL and amphiphilic 1,2-dilauroyl-3-sn-phosphatidylethanolamine (DLPE)-methoxypolyethylene glycol (mPEG) (DLPE-S-S-mPEG) to release doxorubicin in a controlled fashion. Similarly, the other two-component insensitive LPHNPs (ILPNPs) were also prepared from PCL and DLPE-CC-mPEG as controls. The prepared SLPNPs and ILPNPs were around 100–120 nm in diameter with a spherical shape. DOX/SLPNPs and DOX/ILPNPs were both shown to be stable in water and PBS buffer (pH 7.4); however, only DOX/SLPNPs were destabilized under reductive environment, rapidly released DOX to cell nuclei, and exhibited stronger cytotoxicity against cancer cells than DOX/ILPNPs [51].

Photoresponsive techniques are also used to control drug release, either by triggering drug release via the photothermal effects of materials (for example, gold nanorods) or by directly shattering the drug carriers via light irradiation. To create these light-sensitive nanocarriers, the structure should include photochromic groups whose photoreaction might increase polarity and shift the hydrophilic–hydrophobic balance sufficiently to rupture the nanostructure when exposed to light. Yao and colleagues [120] reported a photo-responsive LPHNPs system having a doxorubicin-encapsulated PLGA core coated with a lecithin outer monolayer as an interface and a photo-sensitive layer of a PEG-hexadecyl block polymer with a 2-nitrobenzyl linker with anti-biofouling properties. The photoresponsive polymeric shell triggers drug release under light sensitization. In vitro results showed that the prepared LPHNPs showed excellent light-triggered drug release (76% release with light irradiation versus only 10% release without light irradiation). The confocal microscopy and flow cytometry results also confirmed the light-controlled drug-release behavior of the LPHNs within the cancer cells. 

Redox-responsive LPHNPs are typically intended to release their payload at the tumor site where the concentration of glutathione (GSH) is higher. Wang and colleagues [121] prepared and reported afatinib-loaded, redox-sensitive, Tf-modified LPHNPs (Tf-SS-Afa-LPNs) for selective delivery to lung cancer. The prepared Tf-SS-Afa-LPNs were spherical in shape with a 103.5 ± 4.1 nm particle size and −21.2 ± 2.4 mV zeta potential. The in vitro drug release studies showed an increased afatinib release from 25% to 80% with increasing GSH concentrations from 0.1 to 10 mM, respectively. 

Stimuli-responsive drug release by using external magnetic fields is another fascinating technique. Joshy and colleagues [122] prepared and reported NiFe2O4 NPs (NFO)-reinforced LPHNPs (NLPNs) based on the biodegradable polymer PVA/SA encapsulating zidovudine (AZT). Under the influence of magnetic fields, 80% of the AZT was released and only 13% was released without being subjected to magnetic fields after 48 h due to the presence of magnetic NPs (NFO).

## 7. Patents on LPHNPs

LPHNPs have attracted significant attention owing to their several advantages compared to lipid or polymer NPs. Henceforth, numerous researchers are focusing on translating their research results and are applying to acquire the intellectual property rights. Table 3 presents recently granted patents on LPHNPs.

## 8. Conclusions and Future Perspective

LPHNPs have been reported as a promising nanocarrier class, offering the pharmaceutical benefits and physicochemical characteristics of both polymeric NPs and liposomes. LPHNPs, exhibiting enhanced stability, preventing drug leakage, possessing easy surface functionalization possibilities, and targeting ability, have proven to be profligate carriers, advancing current nanotherapeutics. The surface functionalization of LPHNPs prolongs the blood circulation time, enhances the target specificity, and improves their physicochemical properties as well as their cell associations such as cellular membrane permeability and immune responses. LPHNPs have been reported to successfully deliver siRNA and small molecules individually for cancer therapy while minimizing the toxicity associated with anticancer therapeutics. Although extensive research has been performed in LPHNPs, researchers are advised to focus on exploring the potentially unexplored areas and use the advancements in LPHNPs design to advance the current therapeutics in pharmaceutics. The selection of proper lipid and polymer combinations in the preparation of LPHNPs may improve their performance and long-term stability. Storage stability is another important parameter and decisive factor for their transition from bench to bedside, but it has not yet been established. Furthermore, a global initiative involving researchers, industrial professionals, and the FDA is necessary to address difficulties, bridge the gap, and overcome different impediments to the clinical translation of LPHNPs. To expand the number of products flowing from academic labs to the market, both industry and academia will need to move toward each other and collaborate while keeping regulator expectations in mind.

## Figures and Tables

**Table 1:** Commonly used polymers and lipids for the preparation of LPHNPs with their structures.

Polymer Used	Lipid Used	Reference
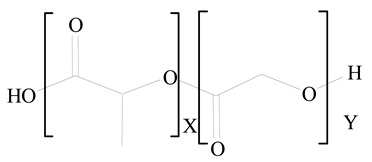 Polylactic-co-glycolic acid (PLGA)	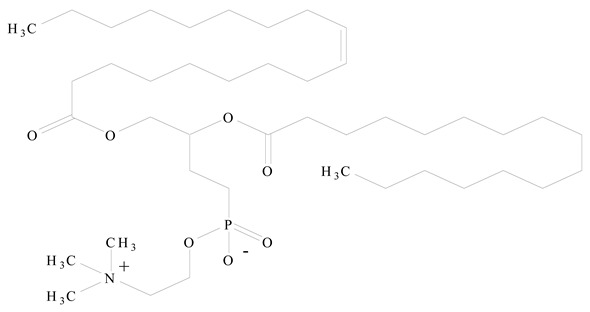 Phosphatidyl choline (lecithin)	[27]
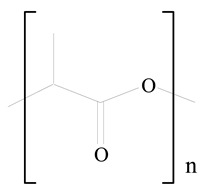 Polylactic acid (PLA)	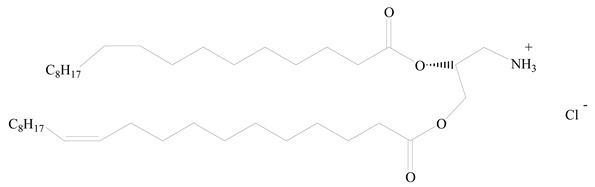 1,2-Dioleoyl-3-trimethylammonium-propane (DOTAP)	[28]
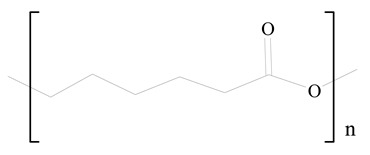 Polycaprolactone (PCL)	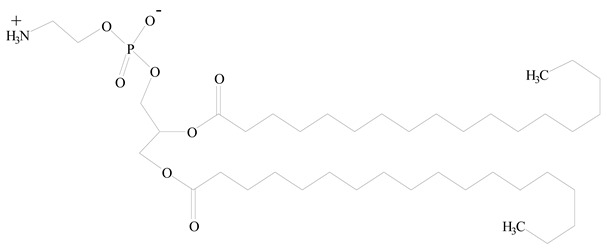 1,2-Distearoyl-sn-glycero-3-phosphorylethanolamine (DSPE)	[29]
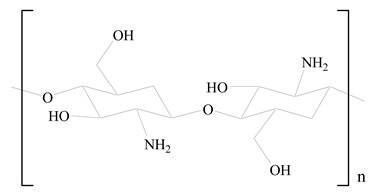 D-Glucosamine and N-acetyl-D-glucosamine (chitosan)	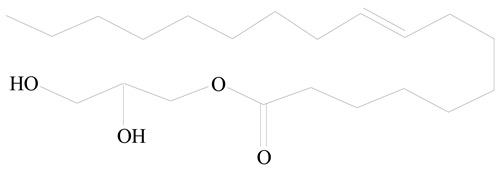 Glyceryl monooleate (GMO)	[30]
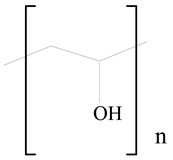 Poly(vinyl alcohol) (PVA)	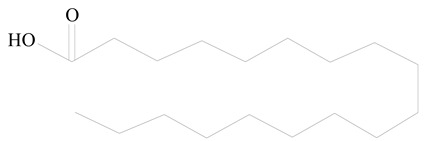 Stearic acid	[31]
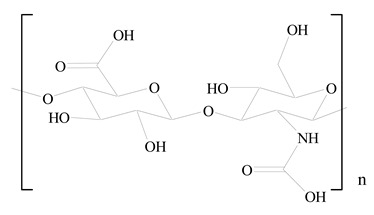 Hyaluronic acid (HA)	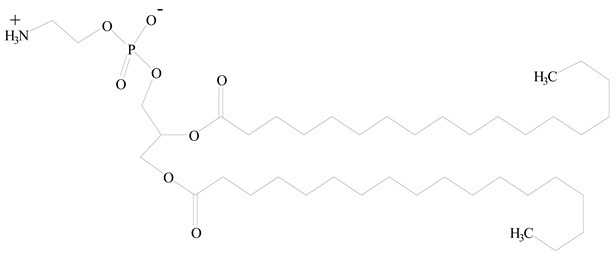 1,2-Distearoyl-sn-glycero-3-phosphorylethanolamine (DSPE)	[32]
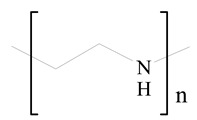 Polyethylenimine (PEI)	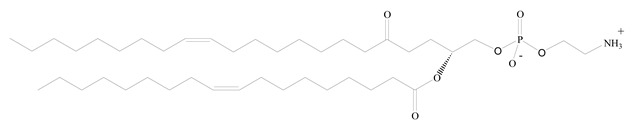 1,2-Dioleoyl-sn-glycero-3-phosphoethanolamine (DOPE)	[33]
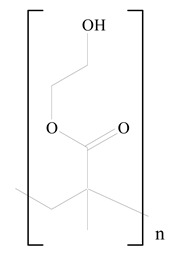 Poly(2-hydroxyethyl methacrylate) (PHEMA)	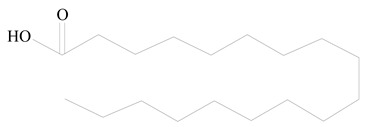 Stearic acid	[34]
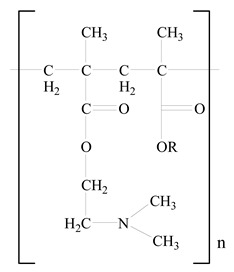 Eudragit	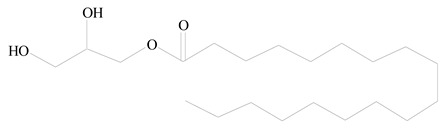 Glycerol monostearate	[35]
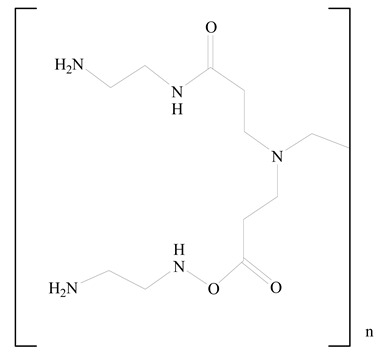 Polyamidoamine	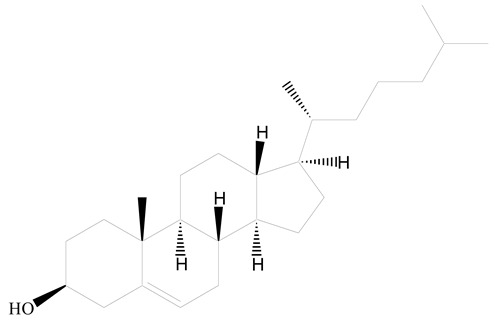 (3β)-Cholest-5-en-3-ol (cholesterol)	[36]
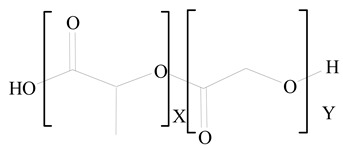 Polylactic-co-glycolic acid (PLGA)	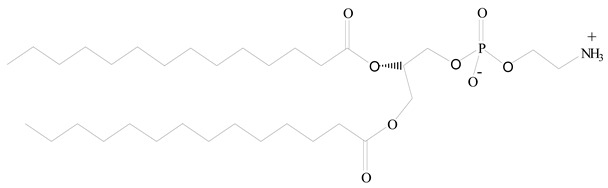 1,2-Dimyristoyl-sn-glycero-3-phosphoethanolaminediethylene (DMPE)	[37]
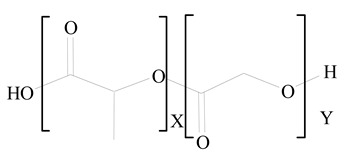 Polylactic-co-glycolic acid (PLGA)	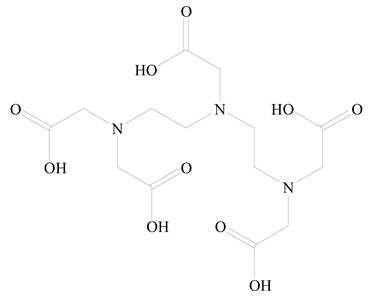 Diethylenetriaminepentaacetate (DTPA)	[37]
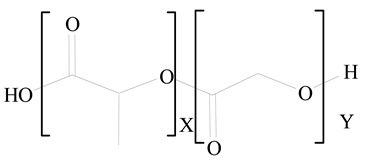 Polylactic-co-glycolic acid (PLGA)	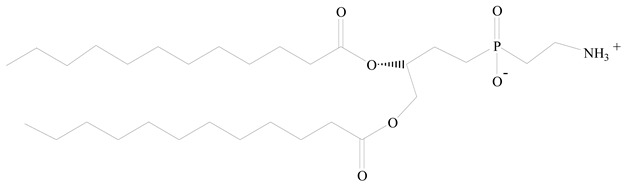 1,2-Dilauroyl-sn-glycero-3-phosphocholine structure (DLPC)	[25]

**Table 2:** Summary of recent published studies on LPHNPs.

Polymer	Lipid	Polymer-to-Lipid Ratio (*w*/*w*)	Method of Preparation	Drugs	Targeting Ligand	Size (nm)	PdI	Zeta Potential (mV)	Application	Reference
PLGA	Lecithin/DSPE-PEG	NR	One step	AuNC/QD	NA	50–100	0.11	NA	Diagnostic/optical imaging	[27]
PLGA	Cholesterol	NR	ESE	CUR	NA	202 and 223	~0.10	−8.0 and −0.6	Cancer therapy	[57]
PLGA	EPC, DOPE	3.5/15	Solvent injection method	7-APTADD	Transferrin	170 ± 7	NR	−18.9 ± 1.5	Cancer therapy (SKBR-3 breast cancer cells)	[58]
PLGA	Lecithin, DMPE, DTPA/ DSPE-PEG	1/0.15	Single-step nanoprecipitation	DTX, 90Y, 111In	A10 Apt	~65	NR	~35	Prostate cancer therapy	[37]
PLGA	Lecithin–DSPE-PEG	1/0.38	Nanoprecipitation process	PTXL and GEM	NA	70 ± 1	NR	−53 ± 2	Cancer therapy (XPA3 pancreatic cancer cells)	[59]
PLGA	Soybean lecithin-DSPE-PEG	9/2.25	-	PTX	NA	~60	NR	NR	Coronary artery disease	[60]
PLGA	DSPE-PEG	1/0.4	Modified nanoprecipitation process	DTX	FA	<25	NR	−10	Cytotoxicity in KB cells (CCL17)	[61]
PLGA	EPC-DSPE-PEG	14/1	Modified double emulsion method	Cy3-siRNA	NA	225 ± 8	NR	−10	Gene delivery for cancer cells (GFP-HeLa cells and of HeLa cells)	[62]
PLGA	PEG-OQLCS-cholesterol	1/1	o/w emulsification solvent evaporation	PTX	FA	184 ± 8	NR	−24 ± 4	Cancer therapy (Hela cervical and A549 lung cancer cells)	[63]
PLGA	DOTAP, DSPE-PEG, cholesterol	NR	Double ESE method	BSA	NA	~144	NR	+4.5–5.6	Vaccine delivery	[64]
PLGA	DOPC, DOTAP, DSPE-PEG	2/15	Spontaneous emulsification and solvent diffusion (SESD) method	Sirolimus or propolis	Fluorescent (NBD-PC lipid and NIR dye–DiD (40 mg))	150–250	0.14–0.26	+ 8 to −33	Cytotoxic studies against human aortic endothelial primary cells (HAECs)	[65]
PLGA	DOTAP	1/0.24	Double ESE method with self-assembly	Plasmid DNA	NA	150–250	0.08–0.17	+36 to +64	Cancer therapy (HEK293T embryonic kidney, HeLa cervical, HaCaT-immortalized human keratinocytes, and HepG2 immortal cancer cells)	[66]
PLGA	RBC membranes	1/1	-	DOX	NA	70–90	0.10–0.25	−10.0 ± 2.7	Leukemia cell line, Kasumi-1	[67]
PLGA	Platelet membranes	5/1	Nanoprecipitation process	DOX, Vancomycin	NA	~115	0.11–0.24	−30.5 ± 0.5	Coronary restenosis	[68]
PLGA	PC/cholesterol/ PEG-DSPE	50/27.5	ESE technique	Combretastatin, DOX	NA	80–120	NR	NR	Cancer therapy (B16/F10 lung cancer cells)	[69]
PLGA	Soybean lecithin-PEG2000, DSPE–PEG2000	40/12	Single-step assembly method	DOX	FA	118 ± 3	0.12 ± 0.01	−8.5 ± 2.4	Cancer therapy (KB oral cavity squamous and COS-7 African green monkey SV40-transformed kidney fibroblast cancer cells)	[70]
PLGA	DLPC/DSPE–PEG	NR	ESE technique	DTX	FA	263 ± 8	0.16–0.03	−20.7 ± 1.2	Cancer therapy (MCF7 breast and NIH/3T3 murine fibroblast cancer cells)	[25]
PLGA	Lecithin, DSPE-PEG	0.3/1	Single-step sonication method	Cisplatin	FA	94 ± 2	0.18 ± 0.06	−19.8 ± 2.4	Cancer therapy (MCF7 breast and A549 lung cancer cells)	[71]
PLGA	DSPE, Lecithin-PEG	1/0.15	Emulsification/solvent diffusion method	DOX	FA	118 ± 0.7	0.10 ± 0.03	15.1 ± 3.8	Cancer therapy (MCF7 breast cancer cells)	[72]
PLGA	EPC/DSPE–PEG	15/1	Modified emulsification technique	10-Hydroxycamptothecin	RGD	249	0.29	−25.6	Cancer therapy (MCF7 and MDA-MB- 435s breast cancer cells)	[73]
PLGA	Lecithin/DSPE–PEG	1/1	Modified single-step nanoprecipitation	Isoliquiritigenin	RGD	137 ± 2	NR	−34.2 ± 1.2	Cancer therapy (MCF7 and MDA-MB- 231 and 4T1 breast cancer cells)	[74]
PLGA	Lecithin/DSPE–PEG	1/0.15	W/O/W ESE process	DTX	RGD	110 ± 13	0.13	−25.6 ± 1.4	Cancer therapy (C6 glioma and GBM brain cancer cell-bearing rats)	[75]
PLGA	DOTAP, DOPE	0.4/0.17	Combination of nanoprecipitation and self-assembly	siRNA	NA	207 ± 4	0.09 ± 0.005	5.2 ± 1.5	Cancer therapy (LNCaP, PC3, and DU145 prostate cancer cells)	[76]
PLGA	DPPC, DSPE-PEG	1/0.2	Single ESE technique	CUR	NA	171 ± 8	0.174 ± 0.02	NR	Cancer therapy (MDA-MB-231 breast and HUVECs human umbilical vein endothelial cancer cells)	[77]
PLGA	Soybean lecithin, DSPE-PEG	1/0.2	Nanoprecipitation method	DOX and 2′-deoxy-5- Azacytidine	NA	80 ± 20	NR	−34.0	Cancer therapy (MDA-MB-231 breast (MB231) and HONE1cancer cells)	[78]
PLGA	Soybean lecithin	2/1	Emulsification-sonication method	PTX, CIS	Arg-Gly-Asp peptide sequence (RGD peptide)	191 ± 5	0.16 ± 0.03	−37.2 ± 3.9	Cancer therapy (A549 lung cancer cells)	[79]
PLGA	Soybean lecithin, DSPE-PEG	2/1	Nanoprecipitation method	PTX, TL	NA	160 ± 5	0.17 ± 0.03	−30.4 ± 4.4	Cancer therapy (A549 and A549/PTX (PTX-resistant) lung cancer cells)	[56]
PLGA	DSPE-mPEG, cholesterol	4/1	Nanoprecipitation technique	Linezolid	NA	50–150	0.15 ± 0.05	−44.0 ± 5.0	Antibacterial activity (osteomyelitis; methicillin-resistant staphylococcus aureus (MRSA))	[80]
PLGA	Lecithin, DSPE-PEG	1/0.2	One-step self-assembly method	Cucurbitacin B	NA	94–112	0.09–0.12	−30.0 ± 5.5	Cancer therapy (MDAMB231 triple-negative human breast cancer cells)	[81]
PLGA	DSPE-PEG	1/0.4	Modified nanoprecipitation process	MK-8722	WYRGRLC peptide	~25	NR	~15	Cartilage targeting for osteoarthritis	[82]
PLGA	DSPE-PEG, HSPC	10/0.1	Modified two-step method	DOX	NA	142 ± 8	0.1	−22.5 ± 4.0	Cancer therapy (M14 DOX-resistant breast cancer cells)	[83]
PLGA	Lecithin, DSPE-PEG	2/0.425	One-step self-assembly method	Lonidamine	NA	110 ± 3	0.17 ± 0.01	−41.2 ± 0.5	Benign prostatic hyperplasia (BPH) treatment	[84]
PLGA	DSPE-PEG	1/0.2	Nanoprecipitation method	DOX and edelfosine	Folic acid	122 ± 2	0.14	−18.4 ± 1.2	Cancer therapy (MG63 bone cancer cells)	[85]
PLGA	Lecithin, cholesterol	15/1	Modified single-step nanoprecipitation self-assembly method	Entecavir (E)	Vitamin E	188 ± 4	0.14 ± 0.02	−21.6 ± 1.0	Antiviral therapy (J774 macrophages cells)	[86]
PLGA	Soy lecithin, DSPE-PEG	NR	Modified nanoprecipitation method	Zinc phthalocyanine and quercetin	NA	170 ± 20	0.30 ± 0.05	−30 ± 10	Cancer therapy (MCF7 breast cancer therapy)	[87]
PLGA	Lecithin, DSPE-PEG	1/0.2	Modified single-step nanoprecipitation process	DOX.HCl	NA	173–208	<0.3	−31.7 to −28.0	Cancer therapy (MDA-MB231 breast and PC3 prostate cancer cells)	[24]
PLGA	DSPE-PEG	1/0.15	Self-assembled nanoprecipitation technique	DTX	NA	143 ± 5	<0.26	−12.2 ± 0.4	Cancer therapy (MDA-MB231 breast cancer cells)	[88]
PLGA	Soy lecithin	1/4	Nanoprecipitation method	PSO	NA	93 ± 2	0.25 ± 0.02	−27.6 ± 0.3	Cancer therapy (MCF7 breast cancer cells)	[89]
PLGA	Soy phosphatidylcholine (SPC), DSPE-PEG	35/65	Modified double ESE method	GEM.HCl	NA	237	<0.3	−16.7 to −24.5	Improve drug encapsulation efficiency and release properties	[90]
PLA	SPC/DPPE/DSPE	1/0.2	Reverse micelle− solvent evaporation technique combined with a self-assembly method	Mitomycin C	FA	215 ± 5	0.14 ± 0.02	−25.8 ± 2.3	Cancer therapy (HeLa cervical and A549 lung cancer cells)	[91]
PLA	DSPE-PEG, SA	1/0.4	Self-assembled nanoprecipitation technique	MTX and BC	Fructose	117 ± 4	0.29 ± 0.11	−6.8 ± 0.2	Cancer therapy (MCF-7 breast cancer cells)	[92]
PLA	DSPC, DOTAP	NR	Surfactant-free solvent diffusion method	LAH4-L1 peptides	NA	151 ± 3	0.09 ± 0.01	−64 ± 3	Cytotoxicity (antigenic presenting cells, namely DC2.4, and epithelial HeLa cells)	[93]
PLA	DOTAP	1/0.1	Double emulsion solvent evaporation method.	EYFP mRNA	Monoclonal anti-CD7	157 ± 9	0.13 ± 0.02	−16.2 ± 0.6	Cancer therapy (A549-CD7 lung cancer cells)	[28]
PCL	DSPE-PEG	2/1	Single-step nanoprecipitation method	MTX and ACL	Fucose	151 ± 5	0.18 ± 0.03	+2.9 ± 0.8	Cancer therapy (MCF7, MDA-MB231 breast cancer cells)	[94]
PCL	DSPE-PEG	2/3	One-step nanoprecipitation	GA and DOX	HA	165 ± 4	0.16 ± 0.02	−41.3 ± 2.8	Cancer therapy (HL-60/ADR leukemia cells and K562/ADR bone cells)	[29]
PCL	SPC	2/5	Sonication method	ERL and BEV	HA	100–120	0.12–0.15	−21.2 ± 2.9	Cancer therapy (A549 and H1975 lung cancer cells)	[95]
PCL	DSPE-PEG, SPC	1/1	Solvent displacement method	CIS and 5-FU	TAB	105 ± 5	0.18 ± 0.20	28.5 ± 1.9	Cancer therapy BE-3 esophageal cancer cells)	[96]
PCL	PEG-DSPE, lecithin	5/1	W/O/W double emulsification method	RPV	NA	112 ± 2	0.16 ± 0.02	−33.2 ± 3.2	Cytotoxicity (BALB/c-3T3 fibroblast viability); In vivo analgesic and anesthesia effect in rats	[97]
Eudragit	SA	2/1	Combined process, using both probe sonication and magnetic stirring processes	DOX	NA	121 ± 3	0.25 ± 0.003	−33.9 ± 3.5	Pharmacokinetic study on healthy rabbits	[98]
Eudragit	Glycerol monostearate, soy lecithin	NR	Modification of the homogenization followed by ultrasonication method	Isradipine	NA	120–124	NR	−28.6	Hypertensive activity in Wistar rats	[35]
Polyamidoamine-grafted cholesterol	DOTAP, DOPE, cholesterol, DSPE-PEG	5/1	Thin-film hydration method	Anti-EGFR siRNA	Peptide (HAIYPRH, named as T7) modified	99 ± 0.6	NR	42.5 ± 1.0	Cancer therapy (MCF7 breast cancer cells)	[36]
Chitosan	DSPE-PEG	NR	ESE method	Sorafenib	Folic acid	178.4 ± 2.6	NR	−21.4 ± 1.5	Cancer therapy (SMMC-7721 liver cancer cells)	[99]
Chitosan	GMO	1/0.2	Self-assembly method	Enoxaparin	NA	~300	<0.3	~20.0	Develop orally applicable delivery system for hydrophilic macromolecules	[30]
Chitosan	Lipoid S75	1/20	Single-step ionic gelation method	CIS	Fluorescent dyes, rhodamine 123, and rhodamine-PE	181–245	0.3–0.4	20–30	Cytotoxicity studies (doxorubicin-resistant A2780 ovarian carcinoma cell line)	[100]
Chitosan	Phospholipon R 80 H	NR	Solvent injection method	Carbamazepine	NA	168 ± 1	NR	−28.9 ± 2.0	Anti epileptic treatment	[101]
Chitosan	Lecithin, DOTAP, DOPE	1/2	Ultrasonication process	AllStar negative-control fluorescent AF488-siRNA	siERK1(mouse) and FITC labeled anti-ERK1 (K-23) mouse	~200	0.18–0.14	41–54	Cytotoxicity studies (NIH3T3 mouse fibroblasts)	[102]
Chitosan	Soy bean lecithin	1.25/1	Syringe method	SLUG mRNA	NA	180	0.2–0.5	20–40	Cancer therapy (MDA-MB453 breast cancer cells)	[103]
PVA	SA	1/0.279	Solvent injection method	Berberine	NA	395 ± 17	0.08 ± 0.01	−18.3 ± 0.1	Antihyperlipidemic activity	[31]
HA	Egg PC, DSPE-PEG	7/2	Solvent evaporation method	Ginsenoside Rg3 (S)-Rg3	NA	134 ± 5	0.24 ± 0.03	−29.5 ± 1.3	Cancer therapy (A549 lung cancer cells)	[32]
PEI- PCL	DOPE, DSPE-PEG	NR	Microfluidic technology	Anti-EGFR siRNA	NA	200 ± 3	0.26 ± 0.04	− 1.2 ± 1.8	Cancer therapy (PC-3 prostate cancer cells)	[33]
PHEMA	SA	4/1	ESE method	CUR	NA	184	NR	−29.3	Cancer therapy (MCF7 breast cancer cells)	[34]
PESO + Pluronic F-68	SA	NR	Ultrasonication method	DOX and GG918	NA	272 ± 48	NR	−19.4 ± 0.3	Cancer therapy (MDA435/ LCC6/MDR1 breast cancer cells)	[104]
PESO	SA, tristearin	NR	ESE method	DOX	NA	290	NR	NR	Cancer therapy (EMT6/WT murine breast cancer cells)	[104]
PCL-PEG-PCL	Soybean, DSPE-PEG	10/1	Thin-film hydration and ultrasonic dispersion method	PTX	FA	279 ± 8	0.17 ± 0.02	−17.5 ± 1.1	Cancer therapy (EMT6 breast cancer cells)	[105]
PLA-PEG-PLA	DDAB	10.8/1.4	Sonication method	FAM-siRNA	NA	48 ± 2	0.25 ± 0.03	12 ± 4	Cancer therapy (MCF7 breast cancer cells)	[106]
pHPMA-chitosan	DSPE-PEG2000; LIPOID S100	NR	Nanoprecipitation	Vitamin B12	NA	135	NR	~18	Mucus penetration and improved cell entry in Caco-2 colon cells	[107]
HPCD	Soy lecithin	NR	ESE method	Amphotericin B and paromomycin	Fmoc-Cl and FITC	164 ± 17	0.39 ± 0.18	−14.7 ± 3.4	Anti-leishmaniasis activity	[108]

Abbreviations: PLGA: Poly(lactic-co-glycolic acid); DSPE: 1,2-Distearoyl-sn-Glycero-3-Phosphoethanolamine; PEG: Poly(ethylene glycol); Folate: FA, AuNC: Silver nanocrystal; NA: Not available; CUR: Curcumin; PC: Phosphatidylcholine; DOPE: 1,2-Dioleoyl-sn-glycero-3-phosphoethanolamine; NR: Not reported; DMPE: 1,2-Dimyristoyl-sn-glycero-3-phosphoethanolaminediethylene; DTPA: Diethylenetriaminepentaacetate; PTXL: Paclitaxel; GEM: Gemcitabine hydrochloride; EPC: Ethylphosphocholine; OQLCS: Octadecyl-quaternized lysine; DOTAP: 1,2-Dioleoyl-3-trimethylammonium-propane; BSA: Bovine serum albumin; DOPC: 1,2-Dioleoyl-sn-glycero-3-phosphocholine; SESD: Spontaneous emulsification and solvent diffusion; NBD: 7-Nitrobenz-2-oxa-1,3-diazol-4-yl; DNA: Deoxyribonucleic acid; RBC: Red blood corpuscles; EPC: Egg phosphatidylcholine; *RGD:* Arginylglycylaspartic acid; DOX: Doxorubicin; TL: Triptolide; DTX: Docetaxel; PSO: Psoralen; PLA: Polylactic acid; SA: Stearyl amine; BC: beta-carotene; MTX: Methotrexate; PCL: Poly-ε-caprolactone; ACL: Aceclofenac; GA: Gallic acid; HA: Hyaluronic acid; ERL: Erlotinib; BEV: Bevacizumab; CIS: Cisplatin; 5-FU: Fluoropyrimidine; TAB: Trastuzumab; RPV: Ropivacaine; GMO: Glyceryl monooleate; PVA: Polyvinyl alcohol; PHEMA: Poly(2-hydroxyethyl methacrylate); PESO: Polymer of epoxidized soybean oil; DDAB: Didodecyldimethylammonium bromide; HPCD: 2-Hydroxypropyl-β-cyclodextrin; Fmoc-Cl: Fluorenylmethoxycarbonyl chloride; FITC: Fluorescein isothiocynate isomer I.

**Table 3:** List of patents granted on LPHNPs for therapeutic applications.

S. No.	Author/Owner	Patent No./Countries Covered	Priority Date	The Title of Invention	Polymer/Lipid Used	Applications
1	Zhang Xueqing, Teng Yilong, Chen Qijing/Rongcan Biomedical Technology (Shanghai) Co., Ltd., China	CN115784920A	9 February 2023	A kind of ionizable lipid compound with high transfection efficiency and its application	PEG/cholesterol, DOPE, and DSPC	Encapsulation of nucleic acids, targeting them to target cells and delivery of nucleic acids of specific genes into cells
2	Song Gengshen, Zhang Honglei, Chen Xichao, Wang Huanyu, Huang Dawei, Yu Xiaowen; Liu Yangjian, Li Yuqing, Yan Rucan, Qiao Lianyong, Li Xiaojuan, Chen Xiaoling, Sun Zhenlong, Wang Shuai, Dong Kai, Zhang Jinyu/Beijing Yuekang Kechuang Pharmaceutical Technology Co., Ltd., China	CN115784921A	8 February 2023	Extrahepatic-targeted cationic lipid compound with high efficiency and low toxicity and its composition	PEG/cholesterol, DOPE	The effective delivery of biologically active substances, including small drug molecules, proteins, peptides, and nucleic acids
3	Yang Hyun-Joo, Wang Jun, Chen Chaoran, Su Miao, Zhang Yuxi, Lin Song, Yu Boya, Du Xiaojiao/Univ South China Tech, China	CN115671045A	30 December 2022	Non-hepatic targeting nucleic acid nano-preparation as well as preparation method and application thereof	PEG-PLA or PLGA/cationic lipids-DOTMA, DOTAP, DORI, DSRIE, DOGS, DOSC.	To regulate genes, proteins, other targets, and the tumor microenvironment
4	Min Peng, Huang Dan, Li Zheng/Keyan Bioengineering Research (Tianjin) Co., Ltd., China	CN115778860A	26 December 2022	Composite of annatto seed oil and hydrogel and its preparation method and application	PGA/annatto seed oil	Cosmetic applications
5	Liu Xuhan, Zhang Jiancheng, Han Wei, Li Qin/Univ Shenzhen General Hospital, China	CN115645523A	22 December 2022	Application of polymer–lipid hybrid nanoparticles as immunologic adjuvant and immune preparation	PEG-PCL, PEG-PLA, PEG-PLGA, PEG-PDL/DOTAP, DOTMA, DDAB, ethyl PC, etc.	As immune adjuvants to improve humoral immunity and cellular immunity, they have advantages in resisting viral infections
6	Liu Qingwei, Tan Bibo, Li Yong, Fan Liqiao, Zhao Qun, Zhang Zhidong, Li Zhaoxing/Fourth Hospital of Hebei Medical University, China	CN115737823A	19 December 2022	A nanodrug delivery system targeting immune cells	DSPC, DMG-PEG/cholesterol, SA	In targeted therapy of tumor immune cells
7	Xu Congfei, Zhang Yue, Wang Yue, Zhao Gui, Wang Jun, Yang Xianzhu/South China University of Technology, China	CN115737841A	7 December 2022	Gene nanomedicine for enhancing T cell anti-tumor immune effect and its preparation method and application	PEG, PLGA/cholesterol derivatives	In chemotherapy and immunotherapy by enhancing the anti-tumor immune effect of T cells for gene nanomedicine
8	Guan Yixin, Zhang Yipeng, Wei Mengying, Liu Xiangrui/Zhejiang University, China	CN115721734A	6 December 2022	Solid lipid nanoparticle complex loaded with budesonide and preparation method thereof	Cellulose, chitosan/SA, monoglyceride stearate, and other fatty acids	The treatment of moderate to severe Crohn’s disease, ulcerative colitis, and other local inflammatory colorectal diseases
9	Ying Bo/Suzhou Abogen Biosciences Co., Ltd., China	WO 2022/152141 A2 CN US	14 January 2021	Polymer conjugated lipid compounds and lipid nanoparticle compositions	PEG (DMG-PEG)/DSPC, cholesterol	The delivery of nucleic acid for therapeutic or prophylactic purposes, including vaccination
10	Kim Yong Hee, Yong Seok Beom, Chung Jee Young, Kim Seong Su, Kim Jae Hyun, Ra Se HeeIucf Hyu, South Korea	US2021379197A1 US, KR	3 June 2020	Dual targeting lipid–polymer hybrid NPs	PLLA, PGA, PLA, PLGA, PCL and PHBV/DSPE, DMPC, DLPC	Applied either alone or in various combination therapies with patient compliance
11	Shah Sunil, Ngu Sean/Max Biology Co., Ltd., USA	WO 2021/234548 A1 IL, CA, AU, US, KR	18 May 2020	Lipid–polymer compositions and methods of use	Poloxamer/PC, phosphatidylserine, phosphatidylglycerol, phosphatidylethanolamine, or phosphatidylinositol	Encapsulation of bioactive agent
12	Cai Yu, Zhuang Yong, Liu Hui, Ma Qianqian, Zhang Ronghua, Yang Li, Wang Panpan, Du Manling, Pang Mujuan/Univ Jinan, China	CN110960509A	30 December 2019	Baicalin polymer–lipid NPs as well as preparation method and application thereof	PEG- 2000, PLGA/distearoylphosphatidylethanolamine	Preparation of breast cancer treatment drugs
13	Chitkara Deepak, Pukale Sudeep Sudesh, Singh Arihant Kumar, Mittal Anupama, Sharma Saurabh/Incisive Element Llc, Nanobrid Innovations Private Limited, India	US 2021/0369631 A1 US, EP, JP	2 February 2019	A lipid–polymer hybrid nanoparticle	mPEG-PLA/Solid lipids (SA, GMS, compritol, precirol, cholesterol or cholic acid), liquid lipid (oleic acid, linoleic acid, miglyol, capmul MCM C8 or captex 355)	The delivery of antibiotics, proteins, peptides, and vaccines
14	Kaczmarek James, Anderson Daniel, Rhym Luke, Kauffman Kevin, Patel Asha/Massachusetts Inst Technology, USA	WO 2020/086965 A9 EP, CA, US, AU	26 October 2018	Polymer–lipids and compositions	PEG/cholesterol	PBAE polymers and formulations
15	Loo Say Chye Joachim, Baek Jongsuep, Tan Chuan Hao/Univ Nanyang Tech, Nat Univ Singapore, Singapore	WO 2019/135715A1 SG	5 January 2018	Lipid–polymer hybrid NPs	PLGA/DOTAP	Lipid–polymer NPs that contain active pharmaceutical ingredient for treating diseases
16	Uehara Keiji, Hatanaka Kentaro, Iwai Hiroto, Naoi Tomoyuki, Destito Giuseppe, Nugent Rachel Soloff/Kyowa Hakko Kirin Co., Ltd., Japan	WO2018225873A1 US, JP	6 June 2017	Nucleic acid-containing NPs	PEG, polyaminoacrylate/polyethylene-glycolated lipids, specifically polyethylene glycol-phosphatidylethanolamine and polyethylene glycol-diacylglycerol, etc.	Nucleic acid-containing NPs that can be delivered to immune system cells
17	Benjamin Frank Geldho/Modernatx, Inc., USA	WO2017223135A1 US	24 June 2016	Lipid NPs	PEG or PEG-DMG, PEG-DSG or PEG-DPG, disteroylphosphatidylcholine, cholesterol	Useful in enhancing the delivery of agents such as nucleic acids
18	Camilla Foged, Henrik Franzyk, Xianghui Zeng, Hanne Mørck Nielsen, Kaushik Thanki/Københavns Universitet, Copenhagen	WO2017158093A1	17 March 2016	Nanoparticle compositions comprising PLGA derivatives and a lipid	PLGA/DOTAP	The delivery of drugs, with enhanced efficacy and reduced adverse effects
19	Pieter Jaap Gaillard, Jacob Rip/Eyesiu Medicines B.V., Netherlands	WO2017025588A1 TW KR RU CN EP US JP	11 August 2015	Pegylated lipid nanoparticle with bioactive lipophilic compound	PEA, PEG/neutral phospholipids comprise at least one of HSPC and DSPE	Systemic or topical delivery of lipophilic diagnostic or therapeutic agents
20	Jacob Klein, Ronit Goldberg, Jasmine Seror, Weifeng Lin, Reut Mashiach/Yeda Research and Development Co Ltd., Israel	US20170128365A1 US CA CN US ES EP ES WO EP	15 June 2015	Surface treatment by water-soluble polymers and lipids/liposomes	PEG/PC (DMPC)	Treating a synovial joint disorder associated with increased articular friction
21	Hatanaka Kentaro, Yagi Nobuhiro, Kuboyama Takeshi, Yagi Kaori, Hosoe Shintaro/Kyowa Kirin Co., Ltd., Japan	US11298326B2	24 March 2015	Nucleic acid-containing lipid NPs	PEG/DSPE, DMPE, cholesterol	As a pharmaceutical and are more stable and smaller than conventional particles
22	Clive Allan Prestidge, Paul Matthew Joyce/University of South Australia, Australia	WO2016141413A1 US CN AU WO	11 March 2015	Drug delivery composition comprising polymer–lipid hybrid microparticles	PLGA/medium-chain triglyceride (MCT; Miglylol^®^812)	The oral delivery of poorly water-soluble drugs, anticancer formulations, and vaccinations
23	Nian Wu/Nian Wu, USA	WO2015085173A1 WO CA US CN JP CN EP	5 December 2014	Polymer–carbohydrate conjugates for drug delivery technology	PEG–carbohydrate conjugates with sterols or “fat-soluble” vitamins (“lipo-vitamin”)	Pharmaceuticals, cosmetics and nutraceuticals
24	Saavedra Steven Scott, Aspinwall Craig A, Ratnayaka Saliya N, Bright Leonard/The Arizona Board of Regents on behalf of the University of Arizona, USA	US 10576456 B2 US	30 June 2014	Systems and methods of preparing stabilized lipid assemblies	Methacrylate, tridecafluoro 1, 1, 2, 2-tetrahydrodimethylchlorosilane (PFDCS)/DPhPC	In ion selectivity, chemical or mechanical gating, inherent signal amplification, well-defined open and closed states and simple electrical readout
25	Geoffrey Gnana Jeba Jesudian, Vijay Kalyansundaram Shastri/Murli Krishna Pharma Pvt. Ltd., India	WO2015136477A1	12 March 2014	NPs of polymer and lipid mixture core for targeted drug delivery	PLGA/GMS, glyceryl behenate	Pharmaceutical drug delivery

Abbreviations: PLA: Polylactic acid; PDL: Polydecalactone; PEG: Poly(ethylene glycol); PGA: Polyglycolic acid; PC: Phosphatidylcholine; DSPC: Distearoylphosphatidylcholine; DDAB: Didodecyldimethylammonium bromide; DORI: Dimethyl-2-hydroxyethyl-2,3-diolene bromide oxypropyl ammonium; DOGS: 1,2-Dioleoyl-3-succinyl-sn-glycerylcholine ester; DOSC: 3β-[N-(N′,N′- Dimethylaminoethyl) carbamoyl] cholesterol; DSRIE: Dimethyl-2-hydroxyethyl-2,3-ditetradecyloxypropyl ammonium bromide; HSPC: Hydrogenated soybean phosphatidylcholine; SA: Stearic acid; DMG-PEG; PLLA: Poly-L-lactic acid; PHBV: Poly(3-hydroxybutyrate-co-3-hydroxyvalerate); DSPE: 1,2-Distearoylphosphatidylethanolamine; DMPC: 1,2-Dimyristoyl-sn-glycero-3-phosphocholine; DLPC: 1,2-dilauroyl-sn-glycero-3-phosphocholine; GMS: Glyceryl monostearate; DPhPC: Methacrylate/1,2-diphytanoyl-sn-glycero-3-phosphocholine; PEA: Palmitoylethanolamide; PBAE: Poly(beta-amino ester); PEG-DSG (1,2-Distearoyl-sn-glycerol, methoxypolyethylene glycol); PEG-DMG (1,2-Dimyristoyl-sn-glycerol) and/or PEG-DPG (1,2-Dipalmitoyl-sn-glycerol, methoxypolyethylene glycol).

## Data Availability

Not applicable.
